# Supplementary material for: Comparative Immunoreactivity Analyses of Hantaan Virus Glycoprotein-Derived MHC-I Epitopes in Vaccination
Source: Vaccines (Basel). 2022 Apr 6;10(4):564. doi: 10.3390/vaccines10040564 (PMC9030823; doi:10.3390/vaccines10040564)
Supplement: Supplementary file 1 [file vaccines-10-00564-s001.zip › Supplementary Figures.pdf]

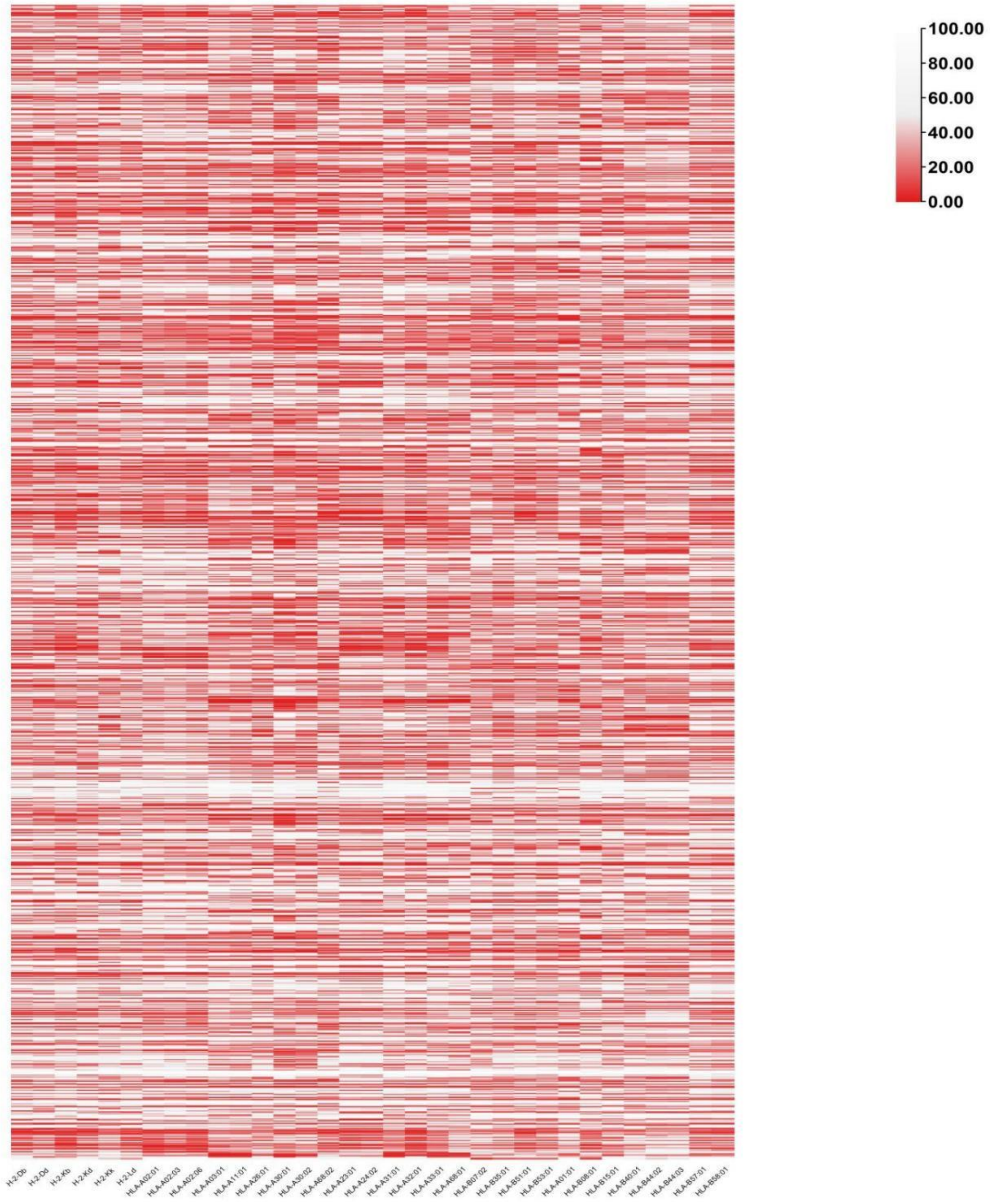

Supplementary Figure S1: Heatmap illustrating the interaction between MHC-I molecules and predicted epitopes. Columns and rows show MHC-I subtypes and epitopes, respectively. Darker colors represent a stronger interaction (lower %rank).

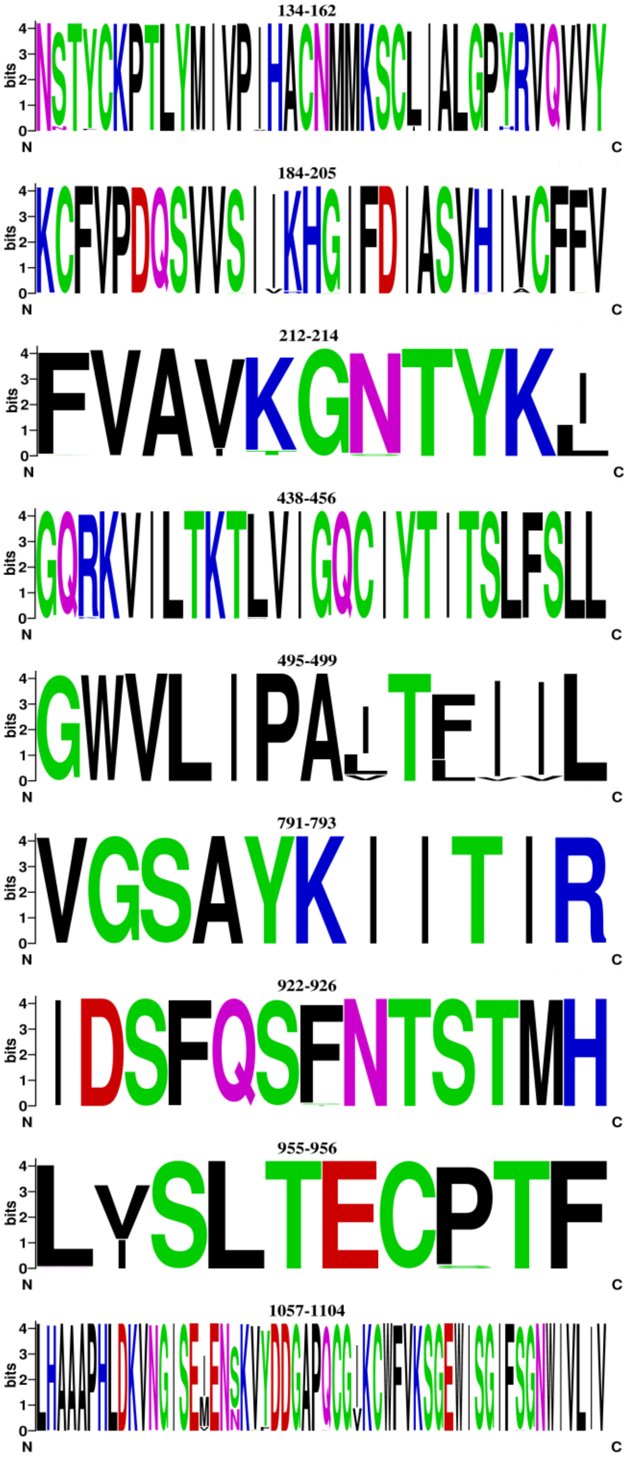

Supplementary Figure S2: Alignment of nine high-affinity segments with the corresponding segments of 148 variants. The overall height of the stack indicates sequence conservation at that position, while the height of symbols within the stack indicates the relative frequency of each a.a. at that position, with a.a.s colored according to their chemical properties as follows: polar (G, S, T, Y, C, Q, and N), green; basic (K, R, and H), blue; acidic (D and E), red; and hydrophobic (A, V, L, I, P, W, F, and M), black.

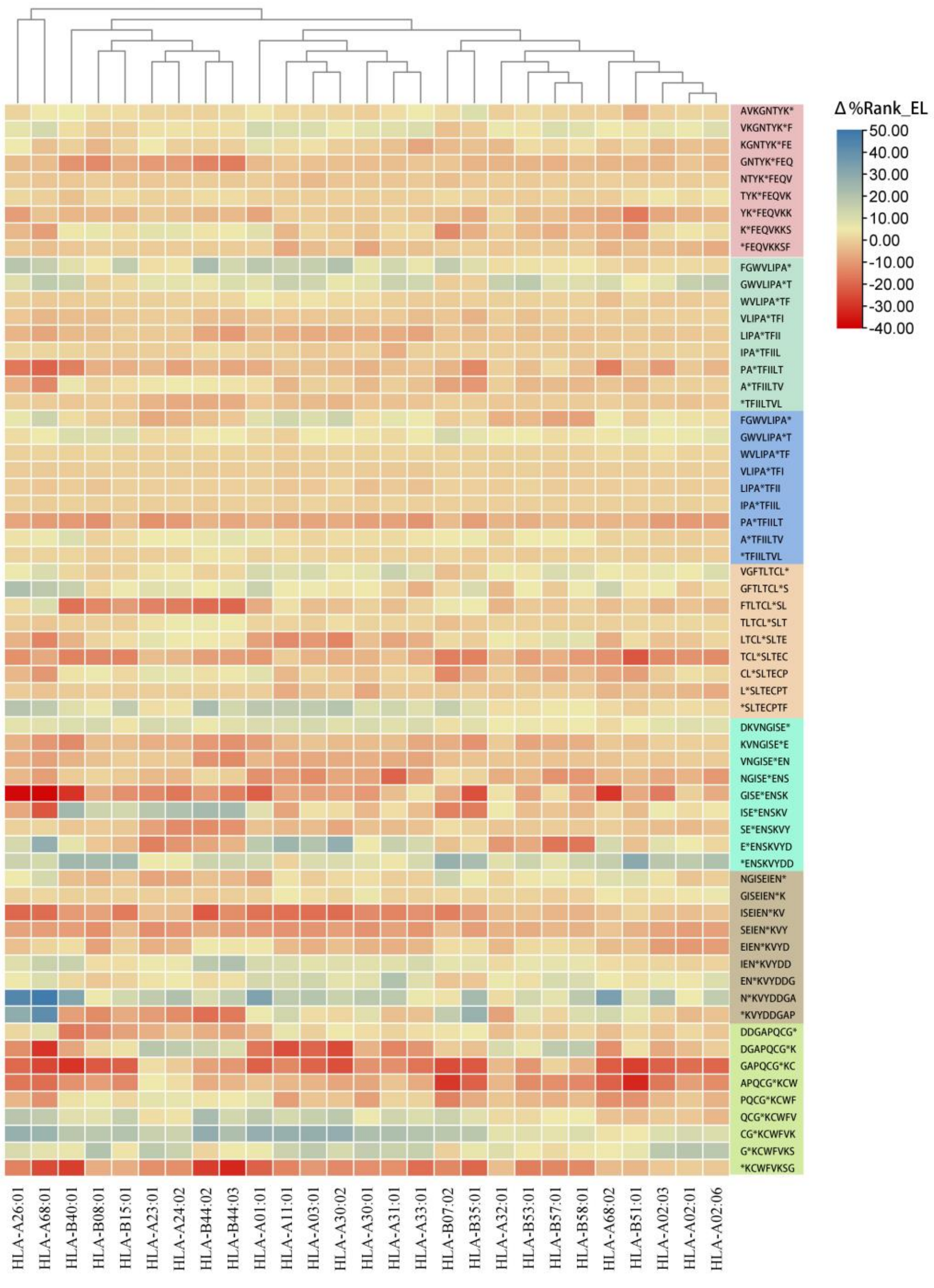

Supplementary Figure S3: The heatmap of 7 consecutive peptide sequences containing each mutation site. TBtools drew the heatmap of the binding affinity delta value between 76-118 and variants of corresponding HLA-I and 9-mer epitopes, where the binding affinity took the logarithm of base 2. The delta value of %Rank as minus represented the epitope of strain 76-118 with better affinity. And contrarily, plus values stood for that of variants acquiring better binding performance with corresponding MHC-I molecule.

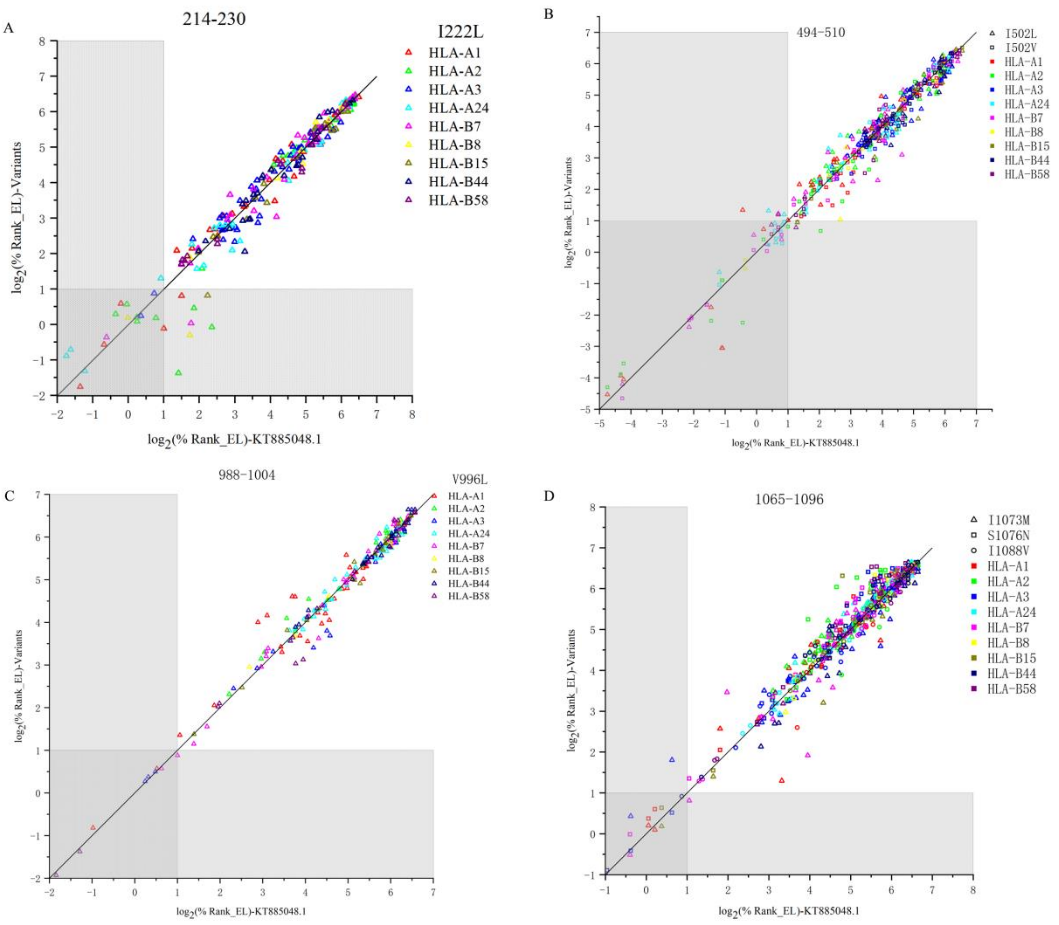

Supplementary Figure S4:

The transverse axis represents the logarithm to the base 2 of the affinity between HTNV 9-mer peptide, and the vertical axis represents the logarithm to the base 2 of that in variants strains. Different colors represent different HLA types and different shapes represent mutations at different sites.
